# Salicylate of Cocaine in the Treatment of Trigeminal Neuralgia

**Published:** 1886-03

**Authors:** 


### Salicylate of Cocaine in the Treatment of Trigeminal Neuralgia.

Schneider (*Allg. med. Chi.-Ztg.*; *Ctrlbl. f. d. ges. Therap.*) relates the case of a woman in her third attack of neuralgia of the second and third branches of the trigeminus. The first attack, five years before, had been treated successfully with large doses of quinine. The second attack lasted almost six months; quinine was of no avail, but the pain gradually disappeared under the use of morphine and iron. The third attack had continued four weeks when the author injected salicylate of cocaine experimentally. The effect was extraordinary; six grains of the salicylate, injected into the cheek, caused the pain to disappear entirely, and occasioned a general feeling of well-being, wholly free from any unpleasant collateral phenomena. The injection itself was painless and did not give rise to irritation. The patient was enabled to sleep at night, although before the pain had been most severe at night. Eight such injections were given in the course of six days, and after that there was no pain, except at the site of the injections, which was overcome by three applications of galvanism with the anode applied to the seat of the pain and the cathode to the back of the neck.—*N. Y. Med. Jour.*

